# Purple stem *Brassica napus* exhibits higher photosynthetic efficiency, antioxidant potential and anthocyanin biosynthesis related genes expression against drought stress

**DOI:** 10.3389/fpls.2022.936696

**Published:** 2022-07-28

**Authors:** Weiqi Chen, Yilin Miao, Ahsan Ayyaz, Fakhir Hannan, Qian Huang, Zaid Ulhassan, Yingying Zhou, Faisal Islam, Zheyuan Hong, Muhammad Ahsan Farooq, Weijun Zhou

**Affiliations:** ^1^Institute of Crop Science, Ministry of Agriculture and Rural Affairs Key Laboratory of Spectroscopy Sensing, Zhejiang University, Hangzhou, China; ^2^Agricultural Technology and Water Conservancy Service Center, Jiaxing, China; ^3^Institute of Crop Science, Zhejiang Key Laboratory of Crop Germplasm, Zhejiang University, Hangzhou, China

**Keywords:** antioxidant enzyme activities, drought, photosynthesis, gas exchange, microscopic study, purple and green stem oilseed rape, reactive oxygen species

## Abstract

Purple-stem *Brassica napus* (*B. napus*) is a phenotype with unique color because of its high anthocyanins content. Anthocyanins are naturally occurring plant pigments that have antioxidants activity and play important role in plant defense against abiotic and biotic stresses. In the present study, drought induced effects on plants were investigated in hydroponically grown seedlings of green stem (GS) and purple stem (PS) genotypes of *B. napus*. The results of this study showed that the major function of anthocyanins accumulation during drought was to enhance the antioxidant capability and stress tolerance in *B. napus* plants. Our results showed that drought significantly inhibited the plant growth in terms of decreased biomass accumulation in both genotypes, although marked decline was observed in GS genotype. The reduction in photosynthetic attributes was more noticeable in the GS genotype, whereas the PS genotype showed better performance under drought stress. Under stressful conditions, both the genotype showed excessive accumulation of reactive oxygen species (ROS) and malondialdehyde (MDA), as well as higher levels of antioxidant enzymes activities. Under drought conditions, the GS genotype showed apparent damages on chloroplast deformation like in thylakoid membrane and grana structural distortion and fewer starch grains and bigger plastoglobuli. Moreover, during drought stress, the PS genotype exhibited maximum expression levels of anthocyanins biosynthesis genes and antioxidant enzymes accompanied by higher stress tolerance relative to GS genotype. Based on these findings, it can be concluded that GS genotype found more sensitive to drought stress than the PS genotype. Furthermore this research paper also provides practical guidance for plant biologists who are developing stress-tolerant crops by using anthocyanin biosynthesis or regulatory genes.

## Introduction

Abiotic stresses are the global challenges that have a significant impact on plant growth and yield attributes (Ahanger et al., 2019). Drought stress has been identified as one of the most severe abiotic stress in the last 40 years than any other natural hazard (Ahanger et al., 2018). Drought is a complex natural phenomenon with varying intensities, durations, regional extents, and consequences. Water deficiency inhibits the cell division, leaf surface, stems growth, cause root cell proliferation, increases reactive oxygen species (ROS) levels and impairs plant growth features (Farooq et al., 2016). Drought is predicted to become more frequent and last longer as a result of global climate change, which may leads to the death of drought-vulnerable plants and have a detrimental impact on global food security (Grieco et al., 2020).

Crop plant exhibits a different range of sensitivity to water stress (Ahanger et al., 2017; Begum et al., 2020). Drought stress damage may affect the opening and closing of plant stomata, osmotic homeostasis, antioxidant system, hormone signal transduction, light protection and metabolic pathway (Shi et al., 2018). Due to the reduction of stomatal closure and CO_2_ diffusion, drought stress reduces the photosynthetic capacity of leaves, lead to ions imbalance, seriously affect the growth and development of plants and reduce crop yield (Ayyaz et al., 2021). Under environmental stressful conditions, photorespiration pathways, photosynthetic machinery and mitochondrial respiration primarily generate the ROS in plant cell. Excess ROS production results in oxidative stress, which disrupts plant cellular metabolism including photosynthetic efficiency, mineral transport and assimilation (Ahmad et al., 2019). To scavenge ROS production, plants activate their antioxidants defense system in the form of enzyme activities such as superoxide dismutase (SOD), peroxidase (POD), catalase (CAT), ascorbate peroxidase (APX), and glutathione reductase (GR) (Khadka et al., 2020). Furthermore, accumulation of secondary metabolites (such as anthocyanins, phenylpropanoids, and terpenoids) boosts antioxidant capabilities and lowers the oxidative stress (He et al., 2017; Ahanger et al., 2019).

Anthocyanins are naturally occurring compounds that are synthesized through biosynthesis of flavonoids. They are responsible for plant pigmentation and grant special colors (including blue, purple and red) to different plant tissues. Anthocyanins are mostly produced in vegetative tissues in response to environmental stress conditions such as low temperatures, pathogens attacks, nutritional deficiency, and heavy metals (He et al., 2020). There are evidences that anthocyanins may protect photosynthetic tissues from photo-inhibition by absorbing blue-green light and therefore limiting the amount of light reaching the chloroplasts (Feild et al., 2001; Neill and Gould, 2003; Hughes et al., 2005; Merzlyak et al., 2008). Flavonoids, like as anthocyanins, are potent antioxidants, raising the possibility that they might scavenge the excess ROS generated during photosynthesis, especially under photoinhibition conditions. Anthocyanins are often present in vacuoles near ROS generation sites (Hatier and Gould, 2008). ROS such as H_2_O_2_ may move rapidly across membranes, various cell compartments, and vacuoles during abiotic stress (Yamasaki et al., 1997; Mittler et al., 2004). Given their antioxidant characteristics, anthocyanins protect the plants against growth retardation and cell death by lowering oxidative stress by scavenging abiotic stress-induced ROS, allowing plants to respond to abiotic stress (Petrussa et al., 2013; Naing et al., 2017; Ai et al., 2018; Wang et al., 2018).

Numerous attempts have been made to increase the anthocyanin in plant tissues (Schwinn et al., 2014). For example, PAP1 gene (*AtPAP1*) is known as the main component of anthocyanin biosynthesis in *Arabidopsis*. The expression of *AtPAP1* genes in tobacco plants resulted in red and purple pigmentations (He et al., 2020). Similarly, transgenic *Brassica napus* plants showed a 50-fold increase in pigmentation including cyanidin and pelargonidin and 5-fold increment in quercetin and sinapic acid (Li et al., 2010). *Brassica napus* is an herbaceous agricultural crop considered as a major cash crop due to its numerous commercial applications such as vegetable oil, animal fodder and biodiesel. In addition, only edible vegetable oil of *B. napus* contributes for about 12% of the global market (Channaoui et al., 2017). Although the green stem color in mustard is related to chlorophyll deposition and purple pigmentation is mostly associated with anthocyanin accumulation. In this study, the new purple stem mustard genotype gives an excellent opportunity to explore the mechanisms of anthocyanins function under drought stress in comparison to green stem mustard genotype. Therefore, we imposed water deficits and evaluated the effect of plant water status on (1) total anthocyanin content, (2) chlorophyll contents and photosystem II efficiency, (3) ROS accumulation and antioxidants mechanism, and (4) the transcriptional regulation of various defense and anthocyanin biosynthesis genes.

## Materials and methods

### Experimental design and growth conditions

Canola (*B. napus*) seeds were obtained from the College of Agriculture and Biotechnology, Zhejiang University, Hangzhou, China. The two genotypes are isolated from segregating populations of the same inbred line and are phenotypically stable after multiple generations of inbreeds. Seeds of these two genotypes were surface sterilized using 0.01% NaClO solution for 15 min followed by vigorous washing with distilled water. The seeds were incubated in darkness for 2 days and placed in growth chamber under control conditions with 16-h photoperiod, the temperature of 24/16°C (day/night) in 300 mM m^−2^ s^−1^ intensity, and 60%–70% relative humidity. After 1 week of seed germination, the seedlings were shifted into one-liter plastic pots filled with half-strength Hoagland nutrient solution (pH 5.8), which contained the following macronutrients: 10 mM NO_3_, 1 mM PO_4_, 6 mM K, 5 mM Ca, 2 mM Mg, and 2 mM SO_4_ and the following micronutrients: 50 μM Fe-EDTA, 230 μM H_3_BO_3_, 3.5 μM Zn, 1.85 μM MoO_4_, 1.6 μM Cu, and 0.7 μM Mn (Dun et al., 2016) for acclimation. Followed by full strength nutrient solution after 1 week of culture. At the third leaf stage, the plants were exposed to drought stress with 10% PEG-6000 solution for 4 days and 8 days. After 8 days of treatment, full strength nutrient solution was used to rewater for 3 days. Each treatment has four pots and each pot has five plants. After treatment, samples were kept in liquid nitrogen and stored at –80°C for further analysis.

### Morphological parameters

After 1 week of drought stress treatments, plants were separated into roots and shoots for biomass measurement. Plants were kept in an oven at 85°C until the seedlings become stable for dry weight determination (Momoh and Zhou, 2001).

### Determination of chlorophyll and gas exchange parameters

The chlorophyll contents were measured by grinding the fresh mature leaf (0.2 g) in 80% acetone at normal room temperature. The mixture was centrifuged at 12,000 rpm for 20 min, and the supernatant was collected for absorbance by spectrophotometer (Hitachi F-4600) according to Arnon (1949).

The photosynthesis rate, stomatal conductance, and transpiration rate of fully matured leaves were evaluated between 9:00 to 11:00 h using a portable photosynthesis system (LI-COR LI-6400XT). Photosynthetic rate (Pn), stomatal conductance (Gs), intercellular CO_2_ concentration (Ci), and transpiration rate (Tr) were determined on the top most fully expanded leaf. The fully expanded leaves were kept in a chamber at 1,000 μmol m^−2^ s^−1^ photon flux density, irradiance 100 w m^−2^ (400–700 nm) with a flow rate at 500 μmol s^−1^ with leaf temperature 28°C. The ambient CO_2_ concentration was kept at 380 μmol CO_2_ mol^−1^ air by keeping the vapor pressure deficit at 2.0 kPa.

### Leaf relative water content (RWC) and electrolyte leakage analysis

The relative water content (RWC) of all the treatments was measured by randomly selecting the mature *Brassica* leaves and fresh weight (FW) was measured. The leaf turgid weight (TW) was measured after dipping the leaves in distilled water for 8 h. Later on, leaves were oven-dried at 70°C for 24 h and then dry weight (DW) was measured (Farooq et al., 2013). The RWC content was calculated by the following formula.

RWC = [(FW − DW) / (TW − DW)] × 100.

Electrolytic leakage was measured by taking fresh leaf discs (1 disc =3 cm × 1 cm) after washing properly. Each leaf disc was added in falcon tube containing deionized water (4 ml). Tubes were shaken for 30 min and EC1 was measured on EC meter. Then, EC2 was measured after boiling the sample for 30 min (Dionisio-Sese and Tobita, 1998).

Membrane stability index = (EC1 / EC2) × 100.

### Chlorophyll fluorescence measurement

A handheld Fluorometer FP-110 (Photon System Instruments, Drasow, Czech Republic) was used to evaluate the photosystem II (PSII) efficiency. Before measurements, samples were put in dark-adaptation for 20 min. PSII fluorescence parameters include the maximum QY (Fv/Fm) of PSII, measured by the following formula Fv = (Fm – Fo). Leaf absorbance for PSI at 830 and 870 nm was balanced first. A weak red light was used to measure Fo and then a saturated pulse of 8,000 μmol m^−2^s^−1^ with 0.8 s width was applied to measure Fm and maximum QY (Fv/Fm) of PSII photochemistry. Various intensities of red actinic light was used which increased stepwise from 0, 11, 18, 27, 58, 100, 131, 221, 344, 536, and830 in 20 s intervals over 5 min. The non-photochemical dissipation of absorbed light energy (NPQ) was measured by the following equation NPQ = [(Fm–Fm′) / (Fm′)]. The gradients of photosynthetic active radiation (PAR) were kept as 0, 100, 200, 300, 400, and 600 μmol m^−2^ s^−1^, respectively (Bilger and Björkman, 1991; Javed et al., 2022).

### Histochemical analyses

The deposition of superoxide (O_2_^−^) and hydrogen peroxide (H_2_O_2_) were visualized by staining the plant leaves with nitro blue tetrazolium (NBT) and 3, 3-diaminobenzidine (DAB). The leaves were kept in dark for 24 h, and ROS accumulation can be visualized in the form of dark and brown spots on leaf surface (Romero-Puertas et al., 2004).

### Membrane lipid peroxidation and reactive oxygen species (ROS) analysis

Malondialdehyde (MDA) is produced by lipid peroxidation and determined by TBA (thiobarbituric acid) reaction (Hong et al., 2010). Leaf tissues (0.5 g) were homogenized with 0.1% TCA (W/V) solution in pre-cooled pistil and mortar. The mixture was centrifuged at 12,000 × *g* for 15 min, and the extract was collected in separate tubes. The reaction solution containing 0.5 ml supernatant, 0.5 ml of 5% TBA and 20% TCA solution incubated in a water bath at 95°C temperature. The reaction stopped by shifting the reaction solution in an ice bath. The absorbance was measured at 532 and 600 nm using spectrophotometer (Hitachi F-4600).

The H_2_O_2_ contents were observed according to the methodology of Chen et al. (2010). Leaf tissues (0.5 g) homogenized with 5 ml of 0.1% TCA (W/V) solution in pre-cooled pistil and mortar. The mixture was centrifuged and extract was collected in separate tubes. The reaction solution contained 0.5 ml enzyme extract, 0.5 ml of 50-mM PBS and 1 ml of 1 M potassium iodide (KI) solution. The reaction stopped by shifting the reaction solution in an ice bath. The absorbance was measured at 390 nm using spectrophotometer (Hitachi F-4600).

### Biochemical assay of antioxidant enzymes, total soluble sugars, and free proline analysis

Leaf samples (0.5 g) were ground in chilled pistil and mortar with 50-mM PBS and centrifuged at 12,000 rpm for 25 min. The extract shifted into another tube for further analysis. SOD assay was determined by following the methodology of Zhang et al. (2008), which depends on SOD capability of NBT inhibition rate of photochemical reduction. The reaction solution comprises of 50-mM PBS, 2-μM riboflavin, 13-mM methionine, 75-μM NBT, 0.1-mM EDTA, and 100-μl enzyme extract making a total volume of 3 ml. The control solution was kept in dark used as blank. One unit activity of SOD is the amount of enzyme required for the inhibition of 50% NBT reduction and measured at 560 nm. CAT activity was assessed by following the methodology of Aebi (1984). POD efficiency was measured following the Chance and Maehly (1955). The reaction solution consists of 50-mM sodium phosphate buffer, 1% guaiacol, 0.4% H_2_O_2_ and 100-μl enzyme extract. The oxidation of guaiacol was assessed at 470 nm. The H_2_O_2_ dependent oxidation rate of ascorbic acid (AsA) was used to measure APX activity, as suggested by Farooq et al. (2016). Glutathione reductase (GR) activity was calculated by the oxidation of NADPH at 340 nm (extinction coefficient 6.2 mM cm^–1^) for 1 min (Islam et al., 2016). Total soluble sugar content was assayed by following the methodology of Fong et al. (1953) and free proline by Bates et al. (1973).

### Ultra-structural observation by transmission electron microscopy

Ultrastructural observations were performed according to Islam et al. (2016). Leaf fragments without veins (about 1 mm^2^) were fixed in glutaraldehyde overnight and then washed three times with PBS. The samples were post fixed in OsO_4_ for 1 h and washed again three times with PBS. After that, the samples were dehydrated in a graded series of ethanol for 15–20 min each and then in absolute acetone for 20 min. After dehydration, the samples were embedded in Spurr’s resin overnight. The specimens were heating at 70°C for 9 h, the ultra-thin sections (80 nm) were cut and mounted on copper grids for the transmission electron microscope (TEM 1230EX, JEOL, Japan) at 60.0 kV.

### Quantitative real-time PCR (RT-qPCR) assay

RT-qPCR a**s**says was followed the method used by Ali et al. (2018), Total RNA was extracted from 100 mg of leaf and root tissues using Trizol (manual) method. Prime script™ RT reagent with genome DNA (gDNA) eraser kit (Takara Co. Ltd., Japan) was used to remove genomic DNA and cDNA synthesis. The synthesized cDNA from different treatment was assayed for quantitative real-time (qRT-PCR) in the iCycleriQTM Real-time detection system (Bio-Rad, Hercules, CA, United States) by using CYBR Premix Ex Taq II (Takara Co. Ltd., Japan).

### Statistical analysis

Data obtained from the current study was the mean (± SE) of at least three replicates. Duncan’s Multiple Range Test was performed to test the significance of data and the least significant difference (LSD) was calculated at *p* < 0.0001 by using the Graph Pad Prism7.

## Results

### Purple sem (PS) maintained higher biomass, relative water content (RWC) and total soluble sugar than green stem (GS) under drought stress

**Figure 1:**
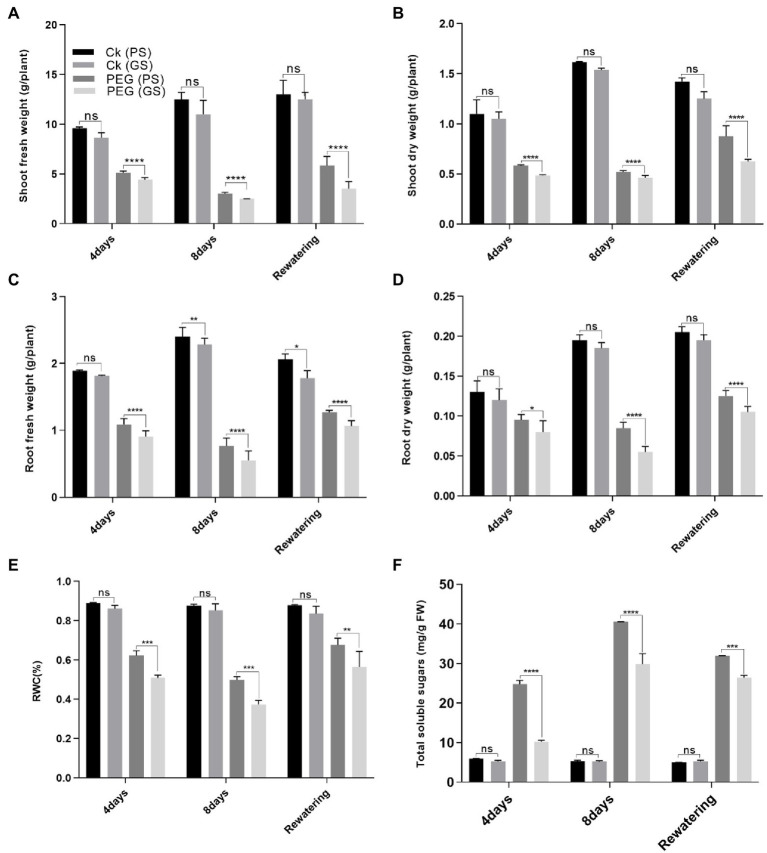
Purple and green stem *Brassica napus* plants growth and morphology under drought (10% PEG-6000), **(A)** shoot fresh weight, **(B)** shoot dry weight, **(C)** root fresh weight, **(D)** root dry weight, **(E)** relative water content (RWC), and **(F)** total soluble sugars, respectively. Different symbols represent significance level as *p = 0.05, **p = 0.01, ***p = 0.001, ****p = 0.0001. ns, non-significance.

Drought stress significantly reduced the plant growth attributes such as shoot and root biomass (fresh and dry weights) and RWC of both PS and GS *B. napus* genotypes compared to control plants (Figures 1A–D). Drought stress reduced (*p* < 0.0001) the shoot fresh biomass of PS genotype by 42% and 78% and GS genotype by 68% and 85% at the 4th and 8th days of PEG treatments respectively, as compared to relative controls (Figure 1A). Similarly, under PEG stress, shoot dry biomass of PS and GS genotypes reduced (*p* < 0.0001) by 39% and 54% at the 4th day, and 48% and 67% at the 8th day, respectively as compared to control plants (Figure 1B). Likewise, root fresh of PS genotype and GS genotype decreased (*p* < 0.0001) by 24% and 41% at the 4th day, and 37% and 57% at the 8th day, respectively, under PEG stress. While, PEG stress reduced root dry biomass by 31% and 46% at the 4th day, and 37% and 61% at the 8th day, respectively compared to control plants (Figures 1C,D). In comparison to controls, the PS genotype showed the greatest increase in shoot fresh biomass by 47% and dry biomass 33% after the re-watering period (Figures 1A–D).

Drought stress reduced (*p* < 0.0001) the RWC content of PS and GS genotypes by 31% and 44%, and 40% and 55%, respectively, after the 4th and 8th days of PEG treatments, as compared to control plants (Figure 1E). Re-watering resulted in in a 10% increase in RWC content of PS genotype when compared to controls. According to our results, the PS genotype was more drought tolerance than the GS genotype, as evidenced by the minimum reduction in fresh and dry biomass and RWC content. Similarly, a considerable increase in TSS content due to PEG enhanced drought tolerance in PS genotype (3- and 7.9-fold change) than GS genotype (1.05-, and 1.4-fold) after 4 and 8 days respectively, as compared to control plants (Figure 1F).

### Purple stem (PS) seedlings accumulated higher chlorophyll contents and photosynthetic efficiency than green stem (GS)

**Figure 2:**
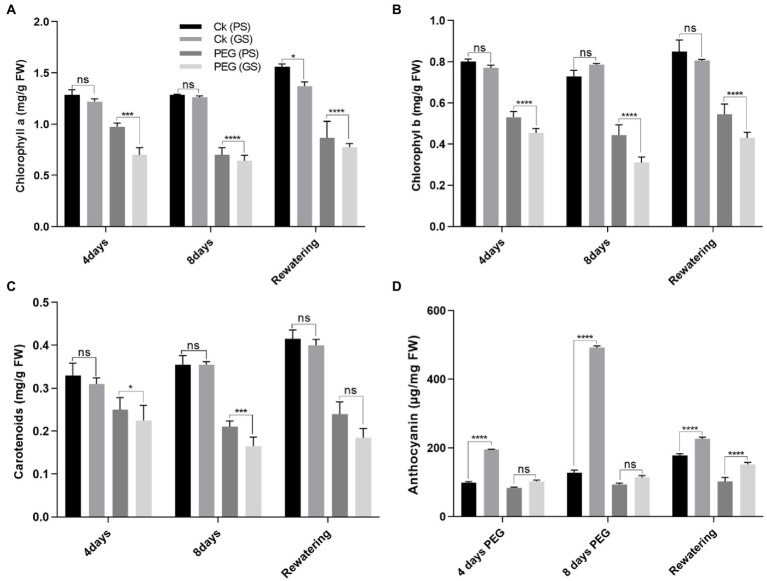
Purple and green stem *Brassica napus* plants under drought (10% PEG-6000), **(A)** chlorophyll a, **(B)** chlorophyll b, **(C)** carotenoids, and **(D)** anthocyanins, respectively. Different symbols represents significance level as *p = 0.05, **p = 0.01, ***p = 0.001, ****p = 0.0001. ns, non-significance.

**Figure 3:**
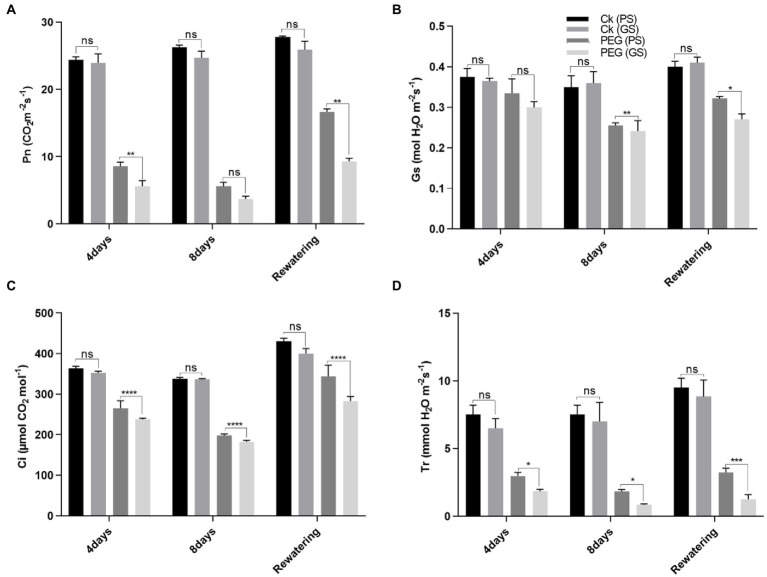
Purple and green stem *Brassica napus* plants under drought (10% PEG-6000), **(A)** net photosynthesis (Pn), **(B)** stomatal conductance (Gs), **(C)** corban dioxide intake (CI), and **(D)** transpiration rate, respectively. Different symbols represents significance level as *p = 0.05, **p = 0.01, ***p = 0.001, ****p = 0.0001. ns, non-significance.

Drought stress reduced chlorophyll production and photosynthetic parameters significantly (*p* < 0.0001) as compared to control. The drought-induced reductions in chlorophyll a of PS and GS genotypes by 18% and 24% at 4th and 31% and 46% at 8th days, respectively, and chlorophyll b by 15% and 19%, and 46% and 62% at 4th and 8th days, respectively, after PEG treatments. While, the carotenoids content of PS and GS genotypes after 4th and 8th days decreased by 15% and 20%, and 26% and 53%, respectively, under PEG treatments (Figures 2A–C). Re-watering on the other hand, resulted in a very slow recovery values for chlorophyll and carotenoids in both genotypes. Anthocyanins accumulation increased in stem, by 31%, in PS genotype after 4th days and 46% after 8th days of PEG treatments (Figure 2D). In GS genotype, the accumulation of anthocyanins was about 13% after 4th days and 18% after 8th days of PEG treatments. However, following re-watering, the PS genotype showed the maximum increase (86%) in anthocyanin among PEG treated plants. Similarly, the decline in Pn rate of PS genotype was about 67% and 80%, and GS genotype was 75% and 86% after 4th and 8th days of PEG treatment respectively, as compared to control plants (Figure 3A). When compared to controls, Gs rate reduced by 26, and 37% in the PS genotype and 36% and 44% in the GS genotype after 4th and 8th days of PEG treatments, respectively (Figure 3B). Relative to control plants, after 4th and 8th days, of PEG treatments, net Ci rate declined by 14% and 23%, and 36% and 39% in PS and GS genotypes respectively, compared to control plants (Figure 3C). Similarly when compared to control plants, Tr rate decreased by 26% and 37% in the PS genotype and 36% and 44% in the GS genotype after 4th and 8th days of PEG treatments, respectively (Figure 3D). Nevertheless, PS seedlings maintained higher photosynthetic pigments and net photosynthetic rate than GS genotype with minimum reduction with and without drought stress compared to controls.

### Maximum quantum yield (QY) of photosystem II (PSII) efficacy of purple stem (PS) seedlings under drought stress relative to green stem (GS)

**Figure 4:**
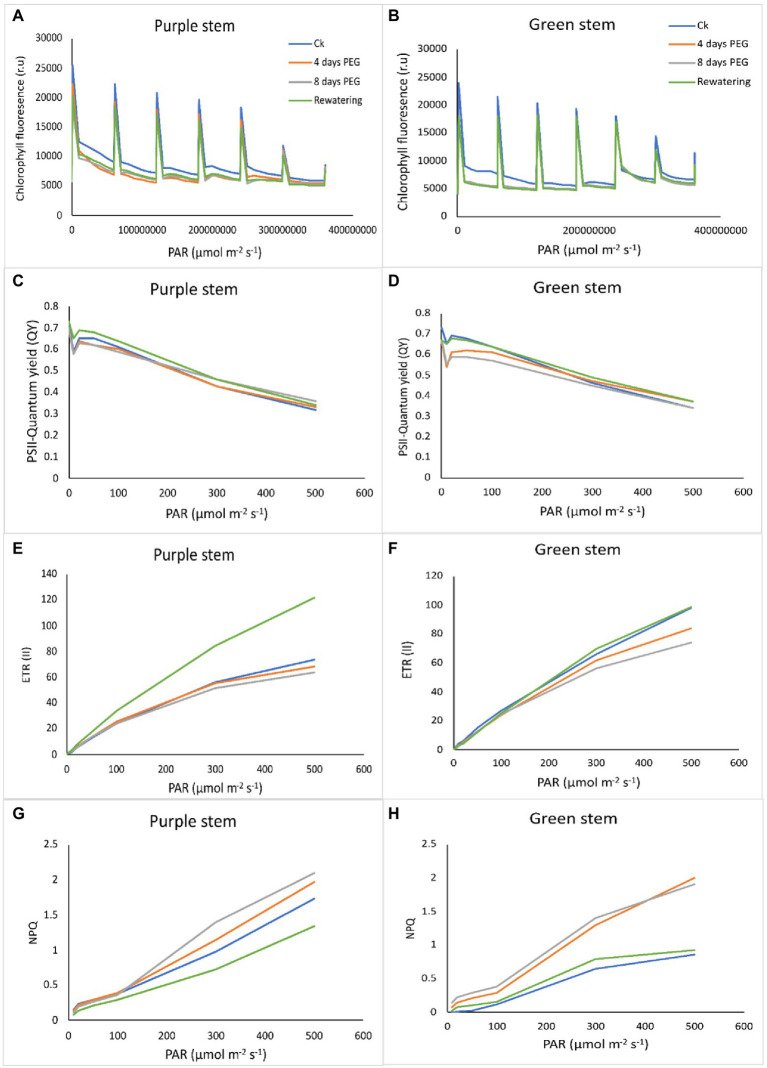
Purple and green stem *Brassica napus* plants under drought (10% PEG-6000), **(A**,**B)** chlorophyll fluorescence (r.u), **(C**,**D)** PSII quantum yield (QY), **(E**,**F)** electron transport rate (ETR II), and **(G**,**H)** non-photochemical uenching (NPQ), respectively.

There is a negative correlation between chlorophyll fluorescence and photosynthetic efficiency. The light energy absorbed by the photosystem can go in different directions: heat dissipation, photochemistry, and fluorescence emission. Measuring the emission of fluorescence light can estimate the proportion of energy flowing to photochemistry. Drought stress remarkably reduced the PSII performance in both *B. napus* genotypes. In the chlorophyll a fluorescence transient, the momentary maximum fluorescence intensity represents the subsequent kinetic bottlenecks of the electron transport chain and depicts the redox state of limitations of plastoquinone (Q_B_). Transients chlorophyll fluorescence of dark adopted leaves under control and drought stress plants disclosed normal light curve (LC1) kinetics in PS that of GS, indicating the better transport of electrons in electron transport chain and redox state of Q_B_. According to our findings regarding the light response curve, in both the genotypes under normal or drought stress, an increase in the electron transport rate through PSII and an increase in non-photochemical quenching are positively correlated with a decrease in PSII efficiency caused by an increase in light intensity (0–800 mol m^−2^ s^−1^; Figures 4A,B). Chlorophyll fluorescence relative values (time based 0–4 × 10^8^ μmol m^−2^ s^−1^) suggested an evident decrease in GS seedlings under drought stress. After 7 days of drought stress, the maximum recovery of PSII activity was observed in PS genotype as compared to GS genotype in re-watering compared to control. Drought stress significantly reduced the quantum yield (QY) of PSII in both *B. napus* genotypes, although this reduction was more apparent in GS than PS relatives to controls (Figures 4C,D). Rapid light curve responsive curve (LC1) analysis disclosed the changes in electron transport ETR (II) and heat dissipation in terms of non-photochemical quenching (NPQ) of both *B. napus* genotypes with and without drought stress. ETR (II) governs the relative electron transport rate (ETR) over PSII, which was declined in both *B. napus* genotypes, ultimately limiting the PSII efficiency. The decrease in ETR (II) was more obvious in GS genotype relative to PS genotype (Figures 4E,F). Under drought stress, NPQ was increased in both PS and GS genotypes, while maximum upsurge was observed in PS genotype than GS genotype (Figures 4G,F). These findings suggested that drought tolerant PS genotypes exhibit greater PSII quantum yield, ETR (II) efficacy and higher photochemical protective mechanisms than drought sensitive GS genotype under drought stress compared to controls.

### Green stem (GS) plants exhibit higher ROS accumulation along with electrolyte leakage (EL) under drought stress

**Figure 5:**
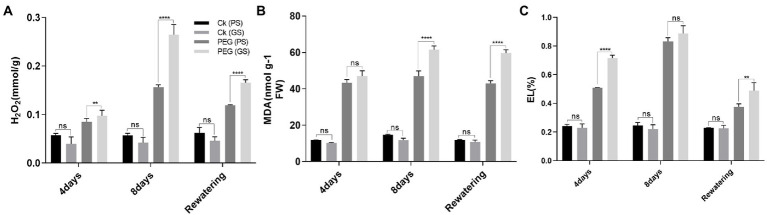
Purple and green stem *Brassica napus* plants under drought (10% PEG-6000), **(A)** hydrogen peroxide (H_2_O_2_), **(B)** malonaldehyde (MDA), and **(C)** membrane stability index: elelectrolyte leakage (EL), respectively. Different symbols represents significance level as *p = 0.05, **p = 0.01, ***p = 0.001, ****p = 0.0001. ns, non-significance.

**Figure 6:**
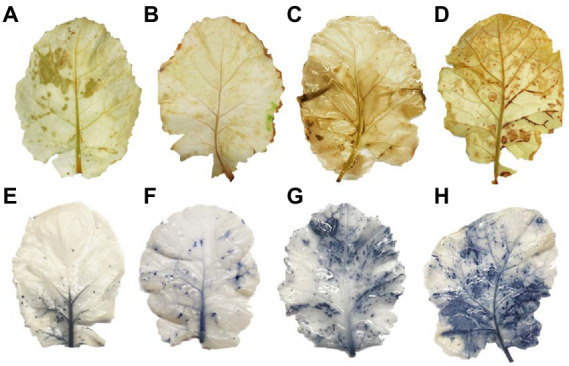
Purple and green stem *Brassica napus* plants growth and morphology under drought (10% PEG-6000) on, respectively. DAB staining **(A)** PS (Ck), **(B)** GS (Ck), **(C)** PS (PEG), **(D)** GS (PEG) and NBT staining, **(E)** PS (Ck), **(F)** GS (Ck), **(G)** PS (PEG), and **(H)** GS (PEG), respectively.

Drought stress remarkably enhanced H_2_O_2_ accumulation, resulting in the lipid peroxidation as MDA and EL in plant cell. PEG treatments on the 4th and 8th days increased H_2_O_2_ and MDA contents by 58% and 94% in PS genotype and 86% and 137% in GS genotype, respectively, as compared to their respective control plants (Figures 5A,B). Similarly the EL of GS genotype was about 76% and 137% at 4th and 8th days under PEG stimulated drought as compared with the PS genotype (34% and 68%; Figure 5C). Furthermore, histochemical analyses with DAB and NBT staining were used to verify the accumulation of ROS (H_2_O_2_ and O_2_^−^). The leaves of both *B. napus* genotypes were stained with and without drought stress conditions (Figures 6A–H). Relative to controls, drought stressed plants displayed brown (H_2_O_2_; Figures 6A–H) and blue (O_2_^−^; Figures 5I–L) staining spots on leaf surface. Under drought stress, DAB and NBT staining revealed that the leaves of the GS genotype exhibits high ROS production and were stained to the deepest extent compare to the PS genotype. While, the least intensity of colored staining of PS genotype suggests that these plants have remarkable efficacy to alleviate the excessive generation and accumulation of ROS under stressful conditions relative to controls.

### Drought tolerant genotype possess higher activities of antioxidant enzymes and proline content under drought stress

**Figure 7:**
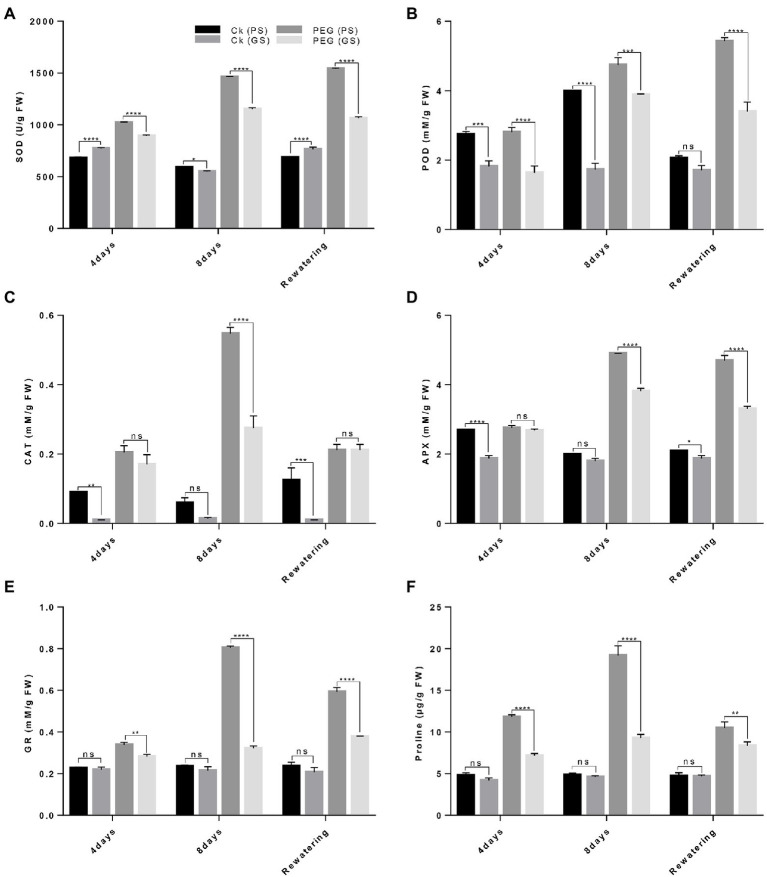
Purple and green stem *Brassica napus* plants under drought (10% PEG-6000), **(A)** superoxide dismutase (SOD), **(B)** peroxidase (POD), **(C)** catalase (CAT), **(D)** ascorbate peroxidase (APX), **(E)** glutathione reductase (GR), and **(F)** proline content of purple and green stem, respectively. Different symbols represents significance level as *p = 0.05, **p = 0.01, ***p = 0.001, ****p = 0.0001. ns, non-significance.

The antioxidant enzymes activity (SOD, POD, CAT, APX, and GR) and proline content varied significantly among both *B. napus* genotypes. Under drought stress, antioxidants activities were considerably increased with higher SOD and POD in PS genotype (96% and 146%) and (68% and 105%) than GS genotype (38% and 58%) and (48% and 89%) as compared to controls plants after the 4th and 8th days of treatments, respectively (Figures 7A,B). Likewise, CAT activity increased by 80% and 195% in the PS genotype and 37% and 66% in the GS genotype after 4th and 8th days, respectively of PEG treatments (Figure 7C). The activities of APX, GR and proline content under drought stress enhanced in PS genotype after at 4th and 8th days (40%, 108%, and 73% and 68%, 147%, and 120%) than of GS genotype (23%, 73%, and 56% and 52%, 88%, and 69%) as compared to control plants, respectively (Figures 7D–F). This indicated that the PS genotype had higher drought tolerance as noticed by the increased antioxidants enzymes activity than GS genotype with and without drought stress.

### TEM reveals differential sub-cellular modifications in *Brassica napus* genotypes under drought stress

**Figure 8:**
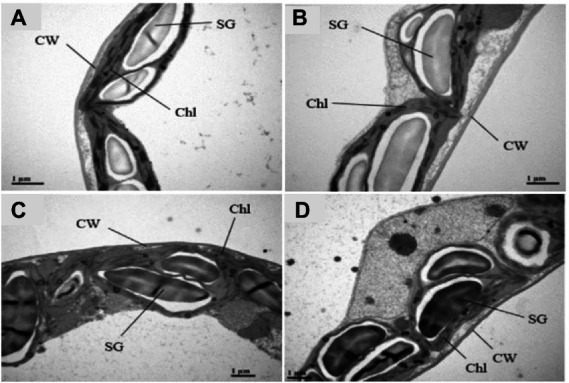
Purple and green stem *Brassica napus* plants under drought (10% PEG-6000), ultrastructure of organelles in mesophyll cells of purple and green stem *B. napus* seedlings under drought and control conditions Chl (Chloroplast), Thy (Thylakoids), CW (Cell wall), SG (Starch grains) of **(A)** PS (Ck), **(B)** PS (PEG), **(C)** GS (Ck), and **(D)** GS (PEG), respectively.

When drought tolerant and sensitive genotypes were compared to controls chloroplast ultra-structures revealed considerable differences in their tissues (Figures 8A–D). Under control conditions, mesophyll cells of the PS genotype showed well-differentiated chloroplasts, mature grana, multiple thylakoid membranes, less plastoglobuli and well-developed stroma lamellae (Figure 8A). The chloroplasts of both the genotypes PS and GS were significantly impacted by drought stress treatment, and chloroplast deformation was obvious. Under drought stress, the chloroplast had fewer disorganized grana and thylakoid membrane with wide intrathylakoid spaces, and small plastoglobuli and decreased number of starch grain as compared to control plants (Figure 8B). Drought stress resulted in visible damage to mesophyll cells of rapeseed plants, although impact was more clear in GS genotype than PS, indicating the higher susceptibility of plants under stressful conditions (Figures 8C,D).

### Drought tolerant genotype showed higher biosynthesis and antioxidant enzymes related gene expression

**Figure 9:**
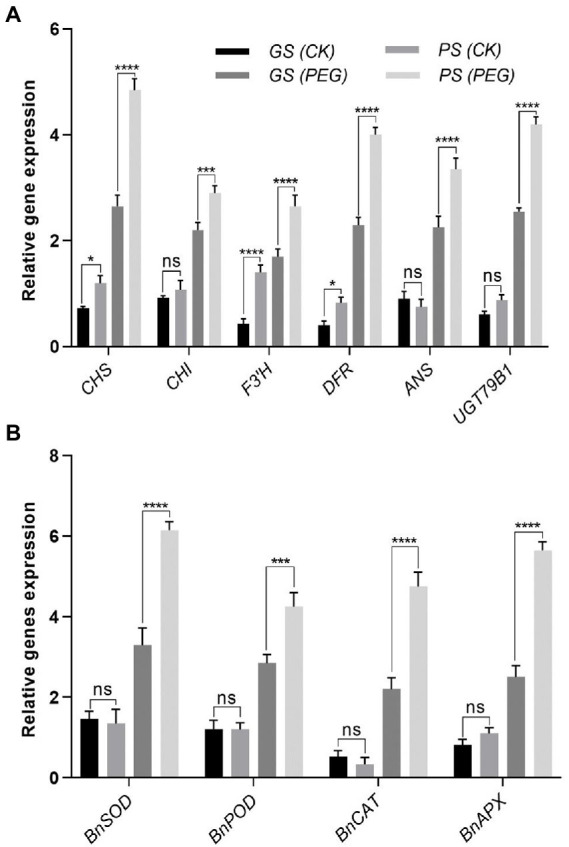
Purple and green stem *Brassica napus* plants under drought (10% PEG-6000). **(A)** Relative expression of anthocyanin biosynthesis genes including *CHS*, *CHI*, *F3’H*, *DFR*, *ANS*, and *UGT79B1*. **(B)** Relative expression of antioxidants enzymes related genes such as *BnSOD*, *BnPOD*, *BnCAT*, and *BnAPX*, respectively. Different symbols represents significance level as *p = 0.05, **p = 0.01, ***p = 0.001, ****p = 0.0001. ns, non-significance.

To explain relative transcript levels of particular genes, qRT-PCR was performed to explain the processes of an expression model for biosynthesis, transcription factors and antioxidant enzyme related genes. Under control and in stressful conditions, the anthocyanin biosynthesis genes including (*CHS*, *CHI*, *F3’H*, *DFR*, *ANS*, and *UGT79B1*) of *B. napus* were differently expressed (Figure 9A). Under drought stress, anthocyanin biosynthesis related genes were significantly expressed as noticed by *CHS* (3.51- and 0.51-fold), *CHI* (2.15- and 0.27-fold), *F3’H* (2.07- and 0.71-fold), *DFR* (1.93- and 1.03-fold), biosynthesis genes *ANS* (2.88- and 1.02-fold), and *UGT79B1* (1.67- and 0.74-fold) were significantly expressed in PS and GS genotypes, respectively relative to controls (Figure 9A). Under drought stress antioxidants enzymes related gene expression level including *BnSOD*, *BnPOD*, *BnCAT*, and *BnAPX* considerably upregulated with a maximum increment of 3.4-, 0.79-, 0.56-, and 2.4-fold and 2.13-, 0.4-, 0.78-, and 1.05-fold in PS and GS genotypes, respectively, compared to controls (Figure 9B). However, the PS genotype exhibited higher expression levels of anthocyanin biosynthesis and antioxidant enzymes related genes relative to GS genotype with and without drought stress, respectively (Figures 9A,B).

## Discussion

Water shortage has a negative impact on agricultural plant development and yield. In the present study, two *B. napus* genotypes (PS and GS) showed considerable genetic variation against drought stress. Based on growth and physio-biochemical attributes, purple stem genotype is considered as drought tolerant and green stem genotype as drought sensitive. Such genetic variability in drought tolerance in *B. napus* genotypes may be due to the differences in physiological and biochemical responses in stressful conditions (Ayyaz et al., 2021). Plant fresh and dry biomass represents growth characteristics and is considered as a key indicator of drought stress assessment. According to our results, drought stress also significantly reduced the plant growth attributes (plant fresh and dry biomass along with RWC) of both *B. napus* genotypes (Figures 1A–F). The reduction was more obvious in drought sensitive GS genotype relative to drought tolerant PS genotype as observed in previous studies on *B. napus*, *Brassica rapa*, and *B. campestris* (Cha et al., 2020; Chen et al., 2020; Grieco et al., 2020; Khanzada et al., 2020; Shawon et al., 2020). Drought stress mediated plant growth reduction is mainly associated with the loss of cell turgor pressure, affected the cell wall biosynthesis and reduced the cell division or cell expansion (Pakdel et al., 2020). These results are quite similar to several studies suggesting that drought stress severely affects the plant growth and RWC in *B. napus*, *B. carinata*, and *Brassica oleracea*, respectively (Dai et al., 2020; He et al., 2020; Samanta et al., 2020). Some previous reports also evaluated the effect of anthocyanin accumulation in wild-type (WT) plants and anthocyanin-enriched transgenic plants that indicated a positive correlation between anthocyanin accumulation and water loss prevention, as well as drought stress tolerance (Nakabayashi et al., 2014; Li et al., 2017a; Naing et al., 2017).

Drought stress reduced the chlorophyll contents and photosynthetic rate along with PSII performance (Khanzada et al., 2020). Plants photosynthetic efficiency is majorly associated with the number of photosynthetic tissues, photosynthetic pigment, CO_2_ influx through stomata, its fixation efficacy and the solar energy conversion efficiency into biochemical energy (Pan et al., 2018; Dąbrowski et al., 2019; Javed et al., 2022). A considerable reduction in photosynthetic pigments and photosynthetic efficacy by drought stress was observed in both *B. napus* genotypes (Figures 2A–D). This might occur due to the enhanced pigments degradation as observed earlier in *B. napus* (Dai et al., 2020). Additionally, the enhanced activity of chlorophyllase enzyme or distortion in thylakoid membrane (Shen et al., 2020) may also involve in inhibiting the photosynthetic efficacy. The anthocyanin rich plants compared with plants lacking anthocyanins may also have the ability to decrease stomatal conductance, which preserves water homeostasis by delaying water loss and wilting during drought stress (Kaku et al., 1992; Choinski and Johnson, 1993).

Reduced photosynthetic performance may also affect the photosystem (PSII) efficiency, which plays significant roles in photosynthetic response against drought stress (Lawlor and Tezara, 2009). Drought stress decreased the quantum yield (QY) of PSII, which is considered a potential indicator of photo-inhibitory damages of PSII (Golding and Johnson, 2003; Kaldenhoff et al., 2007; Lawlor and Tezara, 2009; Bano et al., 2021). Drought stress causes a rapid disassembly of light harvesting complex (LHCII) of PSII. It was observed that short-term and long-term exposure of drought stress makes PSII dimers unstable in *Arabidopsis* seedlings (Chen et al., 2016). This suggests that the assembly of LHCII-PSII super complexes prevents the PSII from photo-damages under drought stress (Chen et al., 2016). The results of our study showed that the QY value of both genotypes leaves was decreased at drought exposure. Comparatively greater reduction in QY values were observed in GS. Zhang et al. (2018) also reported that QY values of the green *Mikania micrantha* were significantly decreased as compared to red *M. micrantha* under low temperature stress. They verified that the accumulation of anthocyanins can improve the plant tolerance under stressful conditions. Similar type of results was also observed in our study where the purple stem *B. napus* plants showed improved performance of photosystem efficiency under drought stress.

The rapid light curve responses revealed that drought stress showed a maximum reduction of PSII efficiency with an increase of light intensity from 0 to 800 μmol m^−2^ s^−1^, which is positively correlates with an increase in electron transport rate (ETRII) and non-photochemical quenching of PSII with and without drought stress in both *B. napus* genotypes (Figures 4E,F). Drought stress induced reduction of ETRII and enhanced NPQ in both the *B. napus* genotypes suggested a considerable accumulation of maximum electrons at PSII acceptor sites that causes an increase in non-photochemical quenching (NPQ; Figures 4G,H). Overall, the reduction in PSII efficiency and ETRII can occur due to reduced size of antenna complex and degradation of LHCII complex proteins. Earlier study also revealed that drought stress severely damaged the LHCII proteins but increased the PsbS proteins (A xanthophyll cycle activating protein of PSII) in *Arabidopsis* seedlings (Chen et al., 2016). Hence, drought tolerant colorful *B. napus* genotype possess higher QY of PSII, ETRII, and NPQ which suggests the maximum photosynthetic performance than green stem seedlings with and without drought stress. Recent studies also showed higher degree of photoinhibition (*Fv/Fm*) in green leaves as compared to the red leaves (Javed et al., 2022).

Drought stress notably increased ROS generation, as measured by MDA, H_2_O_2_, and EL and cause impairments in structural and functional components such as total soluble proteins, lipids and DNA (Zhou and Leul, 1998; Ahmad et al., 2020; Biswas et al., 2020; Rogers, 2020). In the present study, drought sensitive (GS) genotype exhibited higher H_2_O_2_, MDA and EL levels than drought tolerant (PS) genotype against drought stress relative to controls (Figures 5A,B). Several studies provide ample evidences for ROS-induced cellular damage ultra-structural changes and cell death caused by drought stress (Chowardhara et al., 2020; Ray et al., 2020). Our results revealed that drought facilitated membrane lipid peroxidation and electrolyte leakage were declined in drought tolerant *B. napus* genotype (Figures 5B,C). Earlier outcomes also noticed ROS-scavenging, reduction in lipid peroxidation and enhancement in antioxidants enzyme activities in *B. napus*, *Lycopersicon esculentum*, and *Prosopis juliflora* (Mir et al., 2021). This indicates that drought tolerant genotype had strong and efficient antioxidative defense system, which imparts ROS-scavenging process under drought stress. The induction of anthocyanins coupled with increased antioxidant activity may have occurred due to drought-stress-induced ROS signaling, followed by the transcription of anthocyanin-related genes. Nakabayashi et al. (2014) also demonstrated drought-induced anthocyanins accumulation in *Arabidopsis via* transcriptomic and metabolomics analysis. The identified drought-induced anthocyanins were directly associated with oxidative and drought stress tolerance. This may be due to the fact that anthocyanins mainly accumulate in vacuoles close to ROS production sites such as chloroplasts and peroxisomes. Anthocyanins likely scavenge the drought stress-induced ROS that enter the vacuoles, thereby inhibiting chain reactions to avoid excessive ROS accumulation and ensure water homeostasis for plant growth and drought stress tolerance. This association between anthocyanin and drought tolerance was reported in Tobacco (Cirillo et al., 2021) and red and green pretty purple peppers (Bahler et al., 1991). Moreover it is also observed that PS genotype trigger the accumulation of proline and total soluble protein more under drought stress. Mbarki et al. (2018) also reported that the colored wheat genotypes having higher anthocyanin content and proline content under salt stress. It was estimated that high contents of proline and anthocyanin support an active protective response to abiotic stress (Chutipaijit et al., 2011; Sperdouli and Moustakas, 2012).

Plants exposed to environmental stress experienced a disruption in cellular homeostasis, impairing the functioning of cellular components such as chloroplast and cell membranes (Asada, 2006). Ultrastructural analysis revealed that drought exposed plants have enlarged and vesiculated thylakoid membranes of chloroplasts compared to controls. Several researchers also observed ROS-induced chloroplast damage, which was similar to our findings (Sofo et al., 2005; Atkin and Macherel, 2009). Chloroplasts are considered as basic site of ROS generation and severely affected under oxidative stress. ROS scavenging and elimination are promptly required in their site of production before they move to protect the target molecules in thylakoid membrane and stroma of chloroplast (Zhang et al., 2015; Złotek et al., 2015). TEM results suggested that drought stress caused severe damages to structural and functional abnormalities of photosynthetic components under drought stress (Figures 8A–D). While, colorful *B. napus* PS genotype maintained better structural and functional cell integrity compared to green stem GS under same level of drought stress. Similarly, under temperature stress (both heat and chilling), plants synthesize more phenolic compounds such as anthocyanins, flavonoids, and phenolic acids, which ultimately protect the plant cells (Ancillotti et al., 2015; Złotek et al., 2015; Martinez et al., 2016). Anthocyanin application has recently been shown to enhance the ultrastructure of rice seedling under stressful condition. Visualization of leaf cells surface sprayed with anthocyanin showed alleviation of cell shrinkage, mitochondrion and chloroplast damage, and an increase in amounts of endoplasmic reticulum and vesicles (He et al., 2017). Flavonoids biosynthesis occurs by the conversion of 4-coumaroyl CoA to chalcone and naringenin to di-hydro flavonol. Previous studies suggested that *CHS* is considered as the initial rate-limiting enzyme of the flavonoids biosynthesis pathway (Earley et al., 2006; Hichri et al., 2011). Later on, the dihydroflavonol 4-reductase (*DFR*) transfers the dihydroflavonols molecules into colorless leucoanthocyanidins, which are converted into the colored anthocyanidins by anthocyanidin synthase (*ANS*; Falcone Ferreyra et al., 2012). Anthocyanidins after their synthesis glycosylated by UDP-glucose anthocyanidin glucosyltransferase (*UGT79B1*) to form anthocyanins, which further, stabilize through either methylation or acylation (Bhalla and Singh, 2008). The anthocyanins biosynthesis gene expression (*CHS*, *CHI*, *F3’H*, *DFR*, *ANS*, and *UGT79B1*) of purple and green stem plants were significantly enhanced under drought stress when compared with relative controls (Figure 9A). These results were in correspondence with previous studies suggesting that higher anthocyanins accumulation may occurs due to the higher abundance of biosynthesis-related genes (*CHS*, *CHI*, *F3’H*, and *DFR*; Petroni and Tonelli, 2011; Zhang et al., 2014; Gharibi et al., 2019). A significantly enhanced expression of *ANS* and *UGT79B1* genes were noticed under drought stress conditions (Figure 9A), suggesting maximum anthocyanin stability and biosynthesis as compared to control. These transcript levels trigger the promoters of flavonoids metabolic pathways (Kim et al., 2017; Li et al., 2017b; Sun et al., 2017; Naing et al., 2018). The expression of *CHS*, *CHI*, *F3’H*, *DFR*, *ANS*, and *UGT79B1* genes were upregulated in PS than GS with and without drought stress (Figure 9A). This reflects the positive effect on the anthocyanin biosynthesis, which may help plant to cope with various environmental stresses. Overexpression of anthocyanin biosynthesis genes or regulatory TFs in several plant species leads to a higher production of anthoycanins. The anthocyanins scavenge ROS and maintain osmotic balance, thereby increasing abiotic stress tolerance (Wang et al., 2016, 2018; Naing et al., 2017; Ali et al., 2018).

Relative to controls, genes related to antioxidants enzymes such as *BnSOD*, *BnPOD*, and *BnCAT* and *BnAPX* were significantly enhanced with and without drought stress in the PS genotype (Figure 9B). SOD, POD, and CAT encoding genes expression was upregulated along with their enzyme activities in *B. juncea* against high temperature and drought stress (Evaristo de Deus et al., 2015; Saha et al., 2016; Xia et al., 2016; Verma et al., 2021). Naing et al. (2018) also found stress-induced upregulation of antioxidant-related genes (CAT, SOD, and POX) was positively linked with the presence of anthocyanins content (Naing et al., 2018). Drought and salinity stress tolerance in transgenic plants has also been documented due to increased antioxidant and proline activities, which boost water uptake and photosynthesis rate for plant development (Kim et al., 2017; Wang et al., 2016). Similarly, the altered expression of C-repeat binding factor 1 (CBF1) in response to cold stress was observed in the studies of Nakabayashi et al. (2014) and Naing et al. (2018), in which it regulates expression of the two glycosyltransferases genes (UGT79B2 and UGT79B3) involved in modifying anthocyanin metabolic pathways to enhance the abiotic stress tolerance (Nakabayashi et al., 2014). In summary, antioxidants were thoughts to be potentially upregulated in our study as a result of transcriptional and post-transcriptional changes.

## Conclusion

The difference revealed in the current study between purple and green stem *B. napus* shows that both genotypes have different capability to deal with drought stress. Drought severely affected the plant growth attributes, although GS genotype proving to be more sensitive genotype. When exposed to the same level of drought, the colorful PS genotype maintained greater structural and functional cell integrity as compared to green stem GS. The accumulation of anthocyanins in PS genotype under drought stress resulted in improved proline and total soluble sugar contents. Under drought stress, PS plants with increased antioxidant activities reflected the improved ability to reduce excessive ROS generation in cellular organelles. The electron microscopic study indicated that ultrastructural damages in leaf mesophyll cells were more prominent in the GS genotype as compared to the PS genotype under drought stress. Further information is needed on the physiological and metabolic responses observed in color drought-tolerant or resistant species, in order to functionally characterize genes involved in adaptation processes.

## Data availability statement

The original contributions presented in the study are included in the article, further inquiries can be directed to the corresponding authors.

## Author contributions

WC and WZ conceptualized and supervised the work. WC, YM, and AA performed experiments. FH, QH, and ZU helped in analyzing physiological parameters. MF, YZ, and FI provided technical and helpful discussions. AA, ZH, MF, and WZ wrote and edited the manuscript. All authors contributed to the article and approved the submitted version.

## Funding

This work was supported by the Science and Technology Department of Zhejiang Province (2021C02064-2), Collaborative Innovation Center for Modern Crop Production co-sponsored by Province and Ministry (CIC-MCP), and the Agriculture and Rural Affairs Department of Zhejiang Province (2021XTTGLY0202).

## Conflict of interest

The authors declare that the research was conducted in the absence of any commercial or financial relationships that could be construed as a potential conflict of interest.

## Publisher’s note

All claims expressed in this article are solely those of the authors and do not necessarily represent those of their affiliated organizations, or those of the publisher, the editors and the reviewers. Any product that may be evaluated in this article, or claim that may be made by its manufacturer, is not guaranteed or endorsed by the publisher.
